# Dynamic and influential interaction of cancer cells with normal epithelial cells in 3D culture

**DOI:** 10.1186/s12935-014-0108-6

**Published:** 2014-10-30

**Authors:** Laura P Ivers, Brendan Cummings, Funke Owolabi, Katarzyna Welzel, Rut Klinger, Sayaka Saitoh, Darran O’Connor, Yasuyuki Fujita, Dimitri Scholz, Nobue Itasaki

**Affiliations:** School of Medicine and Medical Science, University College Dublin, Dublin, 4 Ireland; Conway Institute, University College Dublin, Dublin, 4 Ireland; School of Biomolecular and Biomedical Science, University College Dublin, Dublin, 4 Ireland; Institute for Genetic Medicine, Hokkaido University, Sapporo, 060-0815 Japan

**Keywords:** 3D culture, Cancer microenvironment, Cell-cell interaction, Time-lapse movie, MDA-MB-231, MCF7, MDCK

## Abstract

**Background:**

The cancer microenvironment has a strong impact on the growth and dynamics of cancer cells. Conventional 2D culture systems, however, do not reflect *in vivo* conditions, impeding detailed studies of cancer cell dynamics. This work aims to establish a method to reveal the interaction of cancer and normal epithelial cells using 3D time-lapse.

**Methods:**

GFP-labelled breast cancer cells, MDA-MB-231, were co-cultured with mCherry-labelled non-cancerous epithelial cells, MDCK, in a gel matrix. In the 3D culture, the epithelial cells establish a spherical morphology (epithelial sphere) thus providing cancer cells with accessibility to the basal surface of epithelia, similar to the *in vivo* condition. Cell movement was monitored using time-lapse analyses. Ultrastructural, immunocytochemical and protein expression analyses were also performed following the time-lapse study.

**Results:**

In contrast to the 2D culture system, whereby most MDA-MB-231 cells exhibit spindle-shaped morphology as single cells, in the 3D culture the MDA-MB-231 cells were found to be single cells or else formed aggregates, both of which were motile. The single MDA-MB-231 cells exhibited both round and spindle shapes, with dynamic changes from one shape to the other, visible within a matter of hours. When co-cultured with epithelial cells, the MDA-MB-231 cells displayed a strong attraction to the epithelial spheres, and proceeded to surround and engulf the epithelial cell mass. The surrounded epithelial cells were eventually destroyed, becoming debris, and were taken into the MDA-MB-231 cells. However, when there was a relatively large population of normal epithelial cells, the MDA-MB-231 cells did not engulf the epithelial spheres effectively, despite repeated contacts. MDA-MB-231 cells co-cultured with a large number of normal epithelial cells showed reduced expression of monocarboxylate transporter-1, suggesting a change in the cell metabolism. A decreased level of gelatin-digesting ability as well as reduced production of matrix metaroproteinase-2 was also observed.

**Conclusions:**

This culture method is a powerful technique to investigate cancer cell dynamics and cellular changes in response to the microenvironment. The method can be useful for various aspects such as; different combinations of cancer and non-cancer cell types, addressing the organ-specific affinity of cancer cells to host cells, and monitoring the cellular response to anti-cancer drugs.

**Electronic supplementary material:**

The online version of this article (doi:10.1186/s12935-014-0108-6) contains supplementary material, which is available to authorized users.

## Background

The tumour microenvironment has a strong influence on cancer cell growth, motility and gene expression [1,2]. The microenvironment comprises extracellular matrix, growth factors, cytokines, oxygen and vasculature as well as the cells that provide them, such as fibroblasts, adjacent cancer cells and/or normal cells.

The effect of normal cells on cancer cells provides a fascinating mechanism for self-defense against cancer. For example, when a single epithelial cell is transformed by an oncogene and is surrounded by normal epithelial cells, the transformed cell is, in many cases, apically extruded and thus eliminated from the epithelium [3,4]. In another example, normal breast epithelial cells or their conditioned media attenuate the growth of co-cultured breast cancer cells [5,6]. In this context, pro-apoptotic factors secreted by the normal epithelial cells are responsible for the attenuation of cancer cell growth [7,8]. In mammary ducts *in vivo*, myoepithelial cells surround luminal cells on their basal side and secrete anti-invasive and anti-angiogenic factors [9]. These examples represent the preventive effect of the normal cellular microenvironment on the early stages of tumour growth and may explain why the chance of a small number of tumour cells successfully establishing a colony or forming secondary tumours in a new location is extremely low [1,10].

Contrary to the preventive effect of the microenvironment, once cancer cells override the self-defense mechanisms and begin to form a tumour, the surrounding cells such as fibroblasts acquire the ability to aid further growth of cancer cells [11,12]. Fibroblasts from breast carcinomas promote the growth of cancer cells more significantly than normal mammary fibroblasts, suggesting co-evolution of cancer and surrounding fibroblasts [13]. This supportive mechanism is seen both in primary locations and in metastatic regions [10].

Despite the importance of the interaction between cancer cells and the surrounding tissues, studies on cellular interaction have been hindered due to difficulties in monitoring actual cellular dynamics *in vivo*. Two-dimensional (2D) *in vitro* culture conditions poorly reflect the *in vivo* cellular behaviour, due to the attachment of cells to the dish. For example, in the ‘homing’ step of metastasis where cancer cells arrive at a new site after extravasation, the interaction of cancer cells with the host epithelium takes place on the basal side of the epithelium [14], which phenomenon cannot be visualised in 2D culture as the cells are attached to the dish. In another scenario, where primary cancer cells grow in the epithelial layer (carcinoma *in situ*), the cancer cells crawl along the gap between the basal side of normal epithelial cells and the basal lamina [3]. Hence, when investigating the interaction of cancer cells with epithelial cells, it is important to make the basal side of the epithelium accessible to the cancer cells; this can only be carried out *in vitro* using 3D cultures. In addition, cellular features such as tumorigenicity and drug resistance are also compromised in the 2D culture method [15,16], thus once again highlighting the benefit of 3D culture in cancer studies.

This study aims to establish a method of 3D culture in conjunction with time-lapse analyses, and to reveal the interaction between breast cancer cells and non-malignant epithelial cells. The breast cancer cell line, MDA-MB-231, a basal-type with highly aggressive features, is mainly used in this study, along with a luminal subtype cell line, MCF7. The cancer cells were co-cultured with normal epithelial cells, either MDCK cells or MCF10A cells, in a reconstituted basement membrane matrix. Time-lapse microscopy revealed the dynamics of the cancer cells on their own and in the presence of normal epithelial cells. Cancer cells are strongly attracted towards the normal epithelial cells and exhibit a destructive behaviour on them when the cancer cells are dominant in the population. However, when the cancer cells are in the minority with a large number of normal epithelial cells, the cancer cells were not destructive toward the epithelial cells despite the continuous interaction. In such conditions, some malignant features in the MDA-MB-231 cells were reduced, as shown by immunocytochemical and cytological analyses. This study reveals the unique interaction of cancer cells with normal epithelial cells from the basal side and highlights the importance of 3D cultures in analysing the dynamics of cancer cells.

## Results

### Morphological diversity and dynamics of MDA-MB-231 cells in 3D

**Figure 1:**
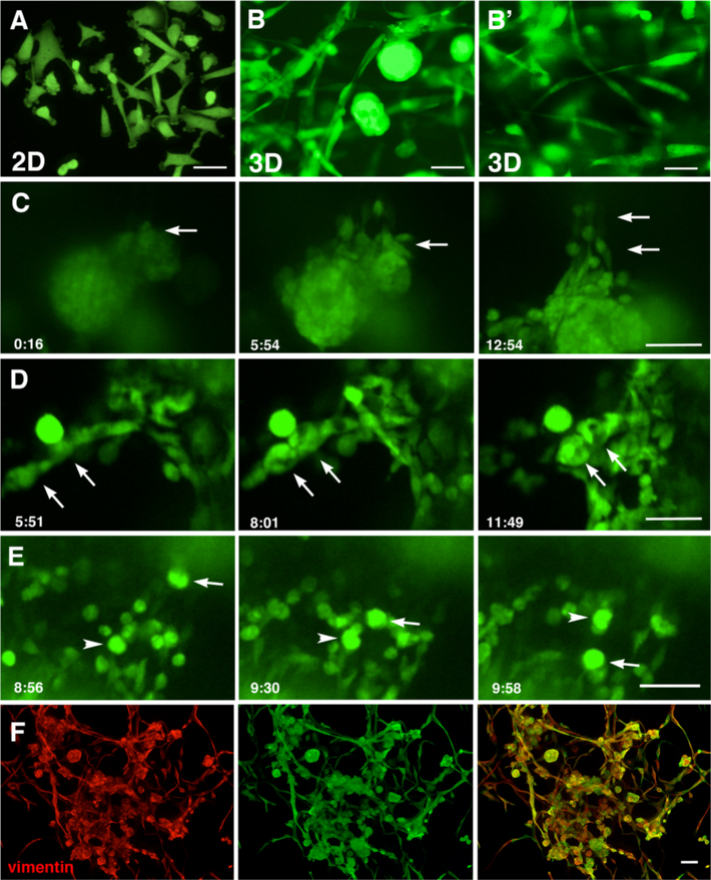
**Dynamic movement of MDA** **-MB** **-231 cells in 3D culture.** All MDA-MB-231 cells are GFP-labelled. **(A-B)** MDA-MB-231 cells cultured in 2D on a plastic dish **(A)** or in 3D in Geltrex matrix (**B** and B’, two examples). In 2D **(A)**, almost all of the cells exhibit spindle-shaped morphology and are dissociated from each other. In contrast, the cells cultured in 3D (**B**, **B’**) are either spindle-shaped or formed aggregates. **(C)** Snapshots of a time-lapse movie (Additional file 1: Movie 1) of MDA-MB-231 cells cultured in 3D, showing the cells in the aggregate dissociating and migrating into the matrix. Arrows indicate cells dissociating from the aggregate. These snapshots show an enlargement of the upper area of Additional file 1: Movie 1. **(D)** Snapshots of a time-lapse movie (Additional file 2: Movie 2) of MDA-MB-231 cells showing elongated cells assembling and forming aggregates. Arrows indicate spindle cells becoming round and forming aggregates. These snapshots show an enlargement of the left-hand side of Additional file 2: Movie 2. **(E)** Snapshots of a time-lapse movie (Additional file 3: Movie 3) of MDA-MB-231 cells showing small aggregates changing location. Two aggregates are indicated by the arrow or arrowhead to follow the translocation. These snapshots show an enlargement of the centre area of Additional file 3: Movie 3. The indicated time in **(C-E)**; hour:min. **(F)** 3D cultured MDA-MB-231 cells immunostained with vimentin (red) and GFP (green), showing that all cells of various cell shapes are vimentin-positive. Scale bars; **A,C-F**, 50 μm; **B** and **B’**, 20 μm.

The breast cancer cells MDA-MB-231 exhibit a spindle-shaped morphology when cultured in 2D [17] (Figure 1A). In contrast, when cultured in 3D, using a reconstituted basement membrane matrix Geltrex®, they either form aggregates as previously described in a similar matrix [18] or distribute in a dissociated manner with various morphologies including very elongated shapes and round shapes (Figure 1B,B’). Therefore, in 3D culture MDA-MB-231 exhibit a wide variety of cell morphologies which are not normally seen in 2D culture. Time-lapse microscopy revealed that their cell morphology is dynamic, such that some cells in an aggregate dissociate and spread into the matrix (Figure 1C; Additional file 1: Movie 1) while others exhibiting elongated shapes assemble and form aggregates (Figure 1D; Additional file 2: Movie 2). The spindle-shaped cells are motile as expected (Additional file 1: Movie 1, Additional file 2: Movie 2). Notably, the round-shaped cells also move very actively as single cells or within the aggregates (Additional file 3: Movie 3). Furthermore, the aggregates themselves are able to move and change location in the matrix while spinning around their centre (Figure 1E; Additional file 3: Movie 3). Both the spindle-shaped cells and those in the aggregates are vimentin positive (Figure 1F), reflecting their motility and active changes in cell morphology [19]. Thus 3D culture reveals the morphological diversity and dynamics of MDA-MB-231 cells.

### Attraction of MDA-MB-231 cells toward normal epithelial cells in 3D culture

**Figure 2:**
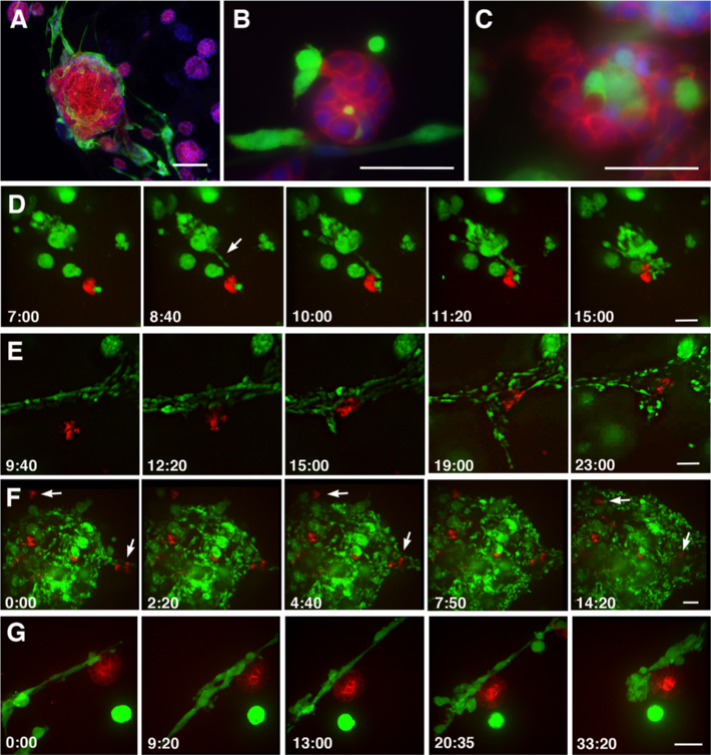
**Dynamic interaction of MDA** **-MB-** **231 and MDCK cells in 3D culture. (A-C)** 3D co-culture of; MDA-MB-231 and MDCK **(A)**, MDA-MB-231 and MCF10A **(B)**, MCF7 and MCF10A **(C)** cells at the ratio of 1:20 for 10 days. The cells were fixed and stained for GFP detecting either MDA-MB-231 or MCF7 cancer cells (green), f-actin (red) highlighting the non-cancerous epithelial cells, and nuclei (blue). In **(A)**, most of the MDA-MB-231 cells are on the surface of a large MDCK mass, which does not show a typical cyst-like structure. In **B** and **C**, a few cancer cells (green) are integrated in the epithelial mass. **(D-G)** Snapshots of time-lapse movies (Additional file 4: Movies 4, Additional file 5: Movies 5, Additional file 6: Movies 6, Additional file 7: Movies 7) showing co-culture of MDA-MB-231 (green) and MDCK (red) cells. In **(D)** (see also Additional file 4: Movie 4), a protrusion of MDA-MB-231 cells (arrow) extends toward the MDCK cells. In **(E)** (see also Additional file 5: Movie 5), the MDCK cells remain static, at a distance from the MDA-MB-231 cells for the first nine hours and are then suddenly attracted towards the stream of MDA-MB-231 cells. Once the MDCK and MDA-MB-231 cells are in contact, the MDA-MB-231 cells rapidly surround the MDCK cells. In **(F)** (see also Additional file 6: Movie 6), several groups of MDCK cells are already in a large mass of MDA-MB-231 cells, however, at the periphery of the MDA-MB-231 mass, a few groups of MDCK cells are seen without MDA-MB-231 contact (0:00 hour time frame; arrows). These MDCK cells become surrounded by the MDA-MB-231 cells by the end of the film. **(G)** (see also Additional files 7: Movie 7) shows an example where there is a relatively small number of MDA-MB-231 cells present near the MDCK cyst. In the first 20 hours of filming, the MDA-MB-231 cells repeatedly attach to and detach from the MDCK sphere. Once several MDA-MB-231 cells begin to gather, they firmly attach to the MDCK sphere. The indicated time; hour:min. Scale bars; 50 μm.

To examine the cellular interaction of carcinoma cells and normal epithelium, GFP-labelled MDA-MB-231 cells were co-cultured in 3D with mCherry-labelled epithelial cells MDCK or MCF10A. When cultured alone in 3D for 7-12 days both MDCK and MCF10A cells form spherical structures (also known as a cysts or acini) displaying clear apical-basal polarity with an internal cavity [20–22]. Spherical structures of MDCK cells without a large clear cavity were also frequently observed in this study, in the culture of less than 12 days (see Figure 2G for example). When co-cultured with MDA-MB-231, the MDCK cells were found either as spheres or as disorganised aggregates, especially when surrounded by many MDA-MB-231 cells (Figure 2A). The MDA-MB-231 cells were not integrated in the mass of MDCK cells. This finding was in contrast to the mix of MDA-MB-231 or MCF7 with MCF10A; normal epithelial cells of mammary gland origin, where masses of MCF10A occasionally included MDA-MB-231 or MCF7 cells (Figure 2B,C). As reported previously, such mixed aggregates exhibit a unique movement of coalescence [6]. For the simplicity of investigating the interaction of cancer cells with the basal side of epithelium, the rest of this study focuses on co-cultures of MDA-MB-231 and MDCK cells. The interaction of MDA-MB-231 or MCF7 with MCF10A cells will be described elsewhere.

**Figure 3:**
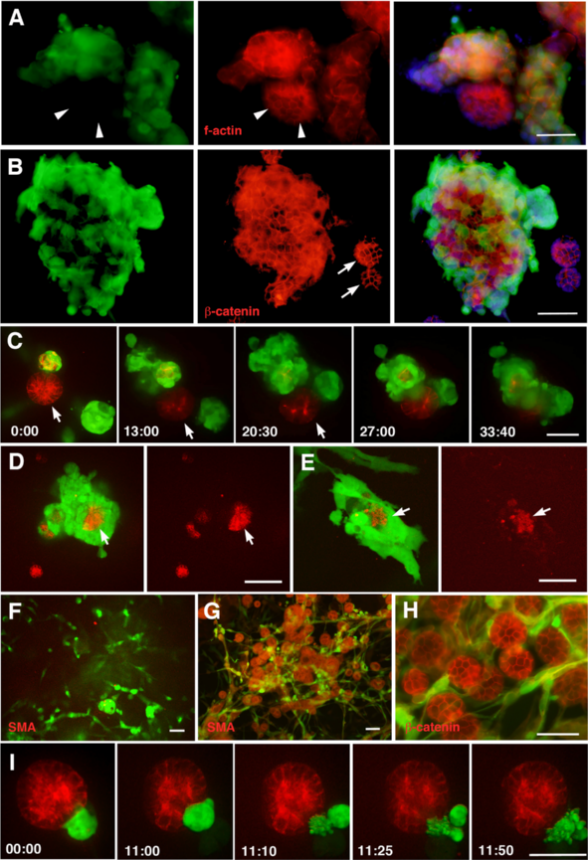
**The mutual effect of MDA** **-MB** **-231 and MDCK cells in a 3D co** **-culture. (A)** Co-culture of MDA-MB-231 and MDCK cells (5:1 ratio), cultured for 8 days and stained for GFP showing the MDA-MB-231 cells (green), f-actin (red) labeling the MDCK cells, and nuclei (blue). The mass of MDCK cells without any MDA-MB-231 cells attached (arrowheads) maintains the typical honey-comb pattern of cytoskeletal organization, whereas the MDCK cell masses that are in contact with MDA-MB-231 cells show disorganised f-actin arrangement. **(B)** A similar culture to A, stained for GFP showing the MDA-MB-231 cells (green), β-catenin (red) highlighting the MDCK cells, and nuclei (blue). The masses of MDCK cells without any MDA-MB-231 cells attached (arrows) maintain the typical honey-comb pattern of cytoskeletal organisation suggesting localisation of β-catenin to the cell membrane, whereas the large MDCK mass with MDA-MB-231 cells attached show β-catenin in the cytoplasm as well as on the cell membrane. **(C)** Snapshots of a time-lapse movie (Additional files 8: Movie 8) showing co-culture of MDA-MB-231 (green) and MDCK (red) cells (5:1 ratio). Throughout the first 25 hours, the MDA-MB-231 cells interact solely with one of the MDCK masses and keep the second MDCK mass intact (arrow). Once a large number of MDA-MB-231 cells fully surround the first mass, they move onto the second MDCK mass (33 h 40 m time frame). **(D)** A snapshot showing co-culture of MDA-MB-231 (green) and MDCK (red) cells (5:1 ratio), cultured in 3D for 11 days. The MDCK cells that are surrounded by MDA-MB-231 cells (arrow) display a debris-like structure. **(E)** A snapshot of Additional file 9: Movie 9 showing MDA-MB-231 and MDCK cells co-cultured in 3D. The MDCK cells (arrow) are surrounded and engulfed by the MDA-MB-231 cells. Some red particles of MDCK debris are seen in the cytoplasm of the MDA-MB-231 cells. **(F)** Snapshots of MDA-MB-231 (green) and MDCK (red) cells (20:1 ratio) co-cultured in 3D for 11 days. The population is now dominated by MDA-MB-231 cells. Only a few pieces of red debris are visible. **(G)** Co-culture of MDA-MB-231 and MDCK cells (1:20 ratio) for 8 days, stained for MDA-MB-231 (green) and for smooth muscle actin (red) mainly labelling MDCK cells. The MDCK cells maintain spherical structures while the MDA-MB-231 cells distribute around them. **(H)** A similar culture to (G), stained for MDA-MB-231 (green) and for β-catenin (red) mainly labeling MDCK cells. MDA-MB-231 cells do not appear to affect β-catenin expression in the MDCK cells. **(I)** Snapshots of a time-lapse movie (Additional file 11: Movies 11) showing a mix of MDA-MB-231 (green) and MDCK (red) cells at a ratio of 5:1 cultured for 11 days in 3D. In this field, there are only a few MDA-MB-231 cells attached to the surface of the MDCK sphere. In the first 11 hours, the MDA-MB-231 cells move while stuck to the surface of the sphere, and then suddenly appear to explode at the 11:00 hour time frame. Scale bars; 50 μm. The indicated time; hour:min.

In MDA-MB-231 and MDCK 3D co-cultures of 8-12 days, many MDA-MB-231 cells were found attached to the surface of MDCK spheres, though not exclusively (Figure 2A, see also Figure 3A,B). Time-lapse microscopy revealed that the MDA-MB-231 cells move continuously whereas the MDCK cells were rather persistent in the form of acini in the 3D culture. The directed movement of MDA-MB-231 cells towards the MDCK cells was observed in the time-lapse analyses which showed protrusion of the MDA-MB-231 cells towards the MDCK cells, even from a distance (Figure 2D, Additional file 4: Movie 4). In other cases, a sphere of MDCK cells was attracted to a stream of MDA-MB-231 cells (Figure 2E, Additional file 5: Movie 5), or a group of MDA-MB-231 cells worked as a mass to restrain the MDCK spheres and surround them (Figure 2F, Additional file 6: Movie 6). As a result of the movement of MDA-MB-231 cells towards the MDCK, most of the MDCK spheres were surrounded by MDA-MB-231 cells after ten days of incubation. The surrounding effect of the MDA-MB-231 cells was prominent when a sufficient number of cells were in the local area. On the contrary, in a low-density culture where a small population of MDA-MB-231 cells was available, the MDA-MB-231 cells continued moving close to the MDCK sphere until a relatively large amount of MDA-MB-231 cells coalesced (Figure 2G, Additional file 7: Movie 7). In summary, MDA-MB-231 cells tend to surround MDCK cells when co-cultured in 3D, and this surrounding activity seems most efficient when a substantial number of MDA-MB-231 cells are present.

### The damaging effect of MDA-MB-231 cells on normal epithelial cells in 3D culture

It was noted that MDA-MB-231 did not attach evenly to all nearby MDCK spheres, i.e. MDA-MB-231 cells attach to only some of available MDCK spheres (Figure 3A,B, see also Figure 2A). Compared to the intact MDCK spheres that lacked attachment of MDA-MB-231 cells, the masses of MDCK cells which had MDA-MB-231 cells attached often exhibited a disorganised cellular arrangement, such as no clear honey-comb-like alignment of cell membrane on the surface of the spheres nor any clear assembly of f-actin on their apical (inner) side (Figure 2A; 3A). A similar disruption of sphere formation was seen in the co-culture of MDA-MB-231 and MCF10A cells (data not shown). The subcellular localisation of β-catenin at the cell membrane was also affected, as cytoplasmic leakage of β-catenin was seen in MDCK cells when MDA-MB-231 were attached to their surface (Figure 3B). Hence, the maintenance of epithelium-specific features is compromised by the attachment of MDA-MB-231 cells. Although, in the fixed cell samples it was unclear as to whether the MDCK cells failed to form spheres due to the attachment of MDA-MB-231 cells, or, whether the MDA-MB-231 cells preferably attached to MDCK cells with a disorganised arrangement. The time-lapse analyses of live cells revealed that the MDA-MB-231 cells tended to approach normal-looking MDCK spheres (Figure 2G; Additional file 7: Movie 7), which in turn triggered the attraction of more MDA-MB-231 cells to the mass. Thereby suggesting that it is in fact the attachment of the MDA-MB-231 cells which causes disruption of the epithelial-specific features in the MDCK acini. Notably, the MDA-MB-231 cells do not begin to surround other nearby MDCK cells until the first-targeted MDCK acinus is fully surrounded by many MDA-MB-231 cells (Figure 3C; Additional files 8: Movie 8). Finally, complete surrounding of MDCK by MDA-MB-231 cells results in destruction of the MDCK cells, whereby the MDCK are seen as debris without any cellular structure (Figure 3D,E. Additional file 9: Movie 9). MDCK-derived small vesicles were seen in the cytoplasm of the MDA-MB-231 cells (Additional file 9: Movie 9).

### The effect of cell ratio and numbers on the behaviour of MDA-MB-231 cells toward MDCK cells in 3D

The above co-cultures were mostly set at the ratio of MDA-MB-231: MDCK = 5:1. Next, a variety of mixing ratios were examined. When the culture was set such that the mix contained twenty-times more MDA-MB-231 cells than MDCK cells (MDA-MB-231: MDCK = 20:1), few MDCK cells were found alive after 10 days of incubation and the remaining cells were mostly seen as debris (Figure 3D-F). This reflects the result of the time-lapse analyses which showed the destructive activity of MDA-MB-231 cells on MDCK (Figure 3E, Additional file 9: Movie 9). On the other hand, when the MDA-MB-231 cells were in the minority (MDA-MB-231: MDCK = 1:20), the MDCK spheres were largely unaffected after 10 days in culture, despite the constant presence of MDA-MB-231 cells migrating on the surface of the MDCK spheres (Figure 3G,H). In the case where the MDCK sphere was large and isolated, MDA-MB-231 cells stayed on the surface of the MDCK sphere for many hours without causing effective destruction, apart from some distortion (Additional file 10: Movie 10). Intriguingly, when a small number of MDA-MB-231 cells stay on the surface of an MDCK sphere without any additional MDA-MB-231 cells joining for hours, the MDA-MB-231 cells appear to break into pieces (Figure 3I; Additional file 11: Movie 11).

These two findings that MDA-MB-231 cells tend to gather on specific MDCK spheres rather than distributing evenly (Figure 2A; 3A-C; Additional file 8: Movie 8) and that the MDA-MB-231 cells are able to destroy the MDCK spheres most effectively when they are in the majority over the MDCK cells (Figure 3D-H), both suggest a community effect, whereby the MDA-MB-231 cells effectively surround and destroy the MDCK cells when there is a sufficient number of them acting as a team. When they work as a team, further MDA-MB-231 cells join and augment the surrounding and destructive effect. However, when the MDA-MB-231 cells are in the minority, they only make transient contacts with the MDCK spheres, and other MDA-MB-231 are not recruited effectively; hence destruction of the MDCK spheres is not successful (Additional files 7: Movies 7; Additional file 10: Movie 10). In the extreme scenario, it appears that the MDA-MB-231 cells stop attacking the MDCK cells when no more MDA-MB-231 cells join them (Figure 3I; Additional file 11: Movie 11). Thus, the ratio of MDCK and MDA-MB-231 cells, as well as the density of the culture, significantly affects; the mode of interaction, expansion of the population and cell survival.

### Ultrastructural analyses of MDA-MB-231 cells co-cultured with normal epithelial cells in 3D matrix

**Figure 4:**
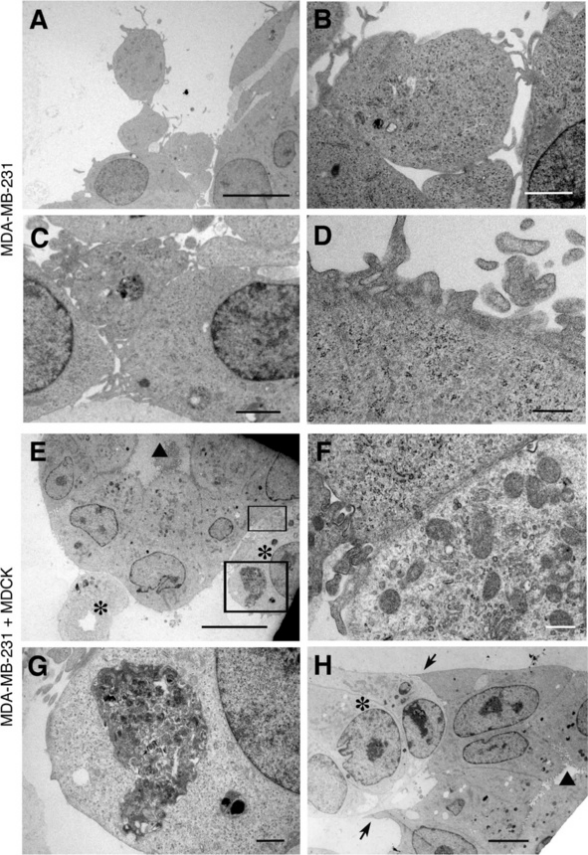
**Transmission electron microscopic images of MDA** **-MB-** **231 cells** **(A-** **D)** **and a 4** **:1 mix of MDA** **-MB-** **231 and MDCK** **(E-** **H)** **after culturing in 3D for 8 days. (A-D)** MDA-MB-231 cells cultured alone show microvilli-like structures on the cell surface. The cells are attached to others only loosely **(B, C)**. The cytoplasm is full of ribosomes, however, organelles such as Golgi apparatus and mitochondria are rarely seen. Scale bars: **A**, 10 μm; **B**, 2 μm; **C**, 2 μm; **D**, 0.5 μm. **(E-H)** MDA-MB-231 co-cultured with MDCK at a ratio of 2:1. The MDCK cells exhibit acinus structures, with numerous microvilli on their apical surface (triangles in **E** and **H**) and the nuclei located on their basal side. The MDCK cells show higher electron density compared to the MDA-MB-231 cells (asterisks in **E**, **H**). **F** is a magnified view of the area shown by one of the rectangles in **E**, where MDCK (upper left) and MDA-MB-231 (lower right) are closely attached. Many mitochondria, ER and Golgi apparatus are seen in the MDA-MB-231 cell. **G** shows another area in the same MDA-MB-231 cell, showing multi-vesicular bodies. **H** shows another example of the attachment of MDA-MB-231 cells (low electron density, the middle one marked with asterisk) to MDCK cells (high electron density, the right-hand-side). While the MDCK cells form an acinus-like structure with microvilli on the apical side (triangle), the basal side is deformed, with sharp processes attached to the MDA-MB-231 cells (arrows). Scale bars: **E**, 10 μm; **F**, 0.5 μm; **G**, 1 μm; **H**, 5 μm.

After filming, the 3D cultures of MDA-MB-231 cells with or without MDCK cells were further processed for transmission electron microscopic analyses, as well as light microscopic analyses. In both transmission electron microscopy (Figure 4E,F,H) and light microscopy using alcian blue staining (not shown), the MDCK cells showed darker cytoplasm compared to the MDA-MB-231 cytoplasm, thus allowing us to distinguish the two types of cells. This was confirmed on sections where MDCK showed typical acinus structures (Figure 4E and not shown).

The MDA-MB-231 cells cultured alone showed loose contacts to each other and many long processes on their surface (Figure 4A-D) [17]. The cytoplasm was full of free ribosomes, with little mitochondria or other organelles (Figure 4A-D), suggestive of high proliferation. When co-cultured with MDCK (MDA-MB-231: MDCK = 2:1), however, the MDA-MB-231 cells had fewer cellular processes in comparison (Figure 4E-H). Mitochondria were prominent in the MDA-MB-231 cells that were in close contact with the MDCK cells (Figure 4E,F). Multi-vesicular bodies were also seen in the cytoplasm of the MDA-MB-231 cells (Figure 4G), which might reflect the phagocyte-like activity seen in Additional file 9: Movie 9.

Most of the MDCK cells maintained apical-basal polarity in the co-culture with MDA-MB-231 cells, with apical microvilli on the luminal side and the nuclei located on the basal side of the cells (Figure 4E,H). However, some of the MDCK cells that were in contact with MDA-MB-231 cells showed sharp processes on the basal surface along the MDA-MB-231 cells (Figure 4H), which were not normally seen when MDCK cells were cultured alone (not shown). This finding may reflect morphological deformity of MDCK cells caused by the contact with MDA-MB-231 cells. Thus the electron microscopic study demonstrates the interplay between MDA-MB-231 and MDCK cells at the subcellular level.

### Cell marker analyses of MDA-MB-231 cells co-cultured with normal epithelial cells in 3D matrix

**Figure 5:**
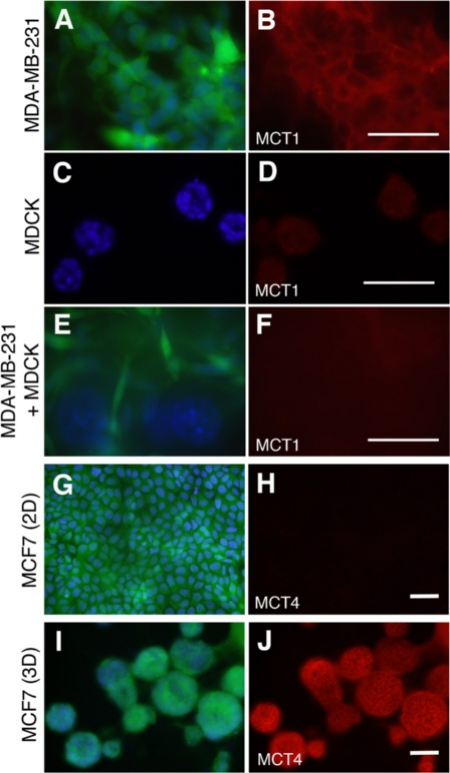
**Expression of monocarboxylate transporters in a co-** **culture of MDA-** **MB** **-231 and MDCK cells and a monoculture of MCF7 in 2D and 3D. (A,B)** MDA-MB-231 cells cultured alone in 3D, stained with anti-GFP (green, A) and anti-MCT1 (red, B) antibodies and with DAPI to visualize the nuclei (blue, A). The majority of MDA-MB-231 cells express MCT1, mostly on the cell membrane. **(C,D)** MDCK cells cultured alone in 3D, stained with anti-MCT1 (red, D) antibody and DAPI to visualize the nuclei (blue, C). The MDCK cells express MCT1, mainly on the cell membrane. **(E,F)** A co-culture of MDA-MB-231 and MDCK cells (1:1 ratio) in 3D, stained with anti-GFP (green, E) and anti-MCT1 (red, F) antibodies and with DAPI to visualize the nuclei (blue, E). E shows a mixed population of MDA-MB-231 (green) and MDCK cells in the sphere (no green). MCT1 is barely detectable in either cell types. **(G,H)** MCF7 cells cultured alone in 2D, stained with anti-GFP antibody labeling MCF7 cells (green, G), anti-MCT4 antibody (red, H) and DAPI to visualize the nuclei (blue, G). The MCF7 cells do not express MCT4 when cultured alone in 2D. **(I,J)** MCF7 cells cultured alone in 3D, stained with anti-GFP antibody for MCF7 (green, I), anti-MCT4 antibody (red, J) and DAPI to visualise the nuclei (blue, I). MCF7 cells strongly express MCT4 on their cell membrane when cultured in 3D. Scale bars; 50 μm.

Immunocytochemical analyses were carried out to identify cellular changes in MDA-MB-231 cells when co-cultured with MDCK cells. MDA-MB-231 and MDCK cells when co-cultured at a ratio of 1:1. At this ratio, a substantial amount of MDCK cells kept growing for more than a week without significant cell death, thus increasing the likelihood of interaction of the MDA-MB-231 cells with the MDCK cells. Among tested markers, a prominent change was observed in the expression of monocarboxylate transporter-1 (MCT1). MCTs are a family of plasma membrane proteins which function to transport monocarboxylates such as lactate and pyruvate between neighbouring cells, and are involved in the metabolism of cancer and the surrounding tissue [23,24]. Most cancer cells generate energy by glycolysis and produce monocarboxylates that are used to synthesise macromolecules required for rapid cell proliferation. In advanced cancer where the microenvironment promotes the growth of cancer, cancer cells intake additional lactate molecules from the surrounding cells. This increase in lactate uptake is manifested by changes in the expression of MCTs. MCT1 is elevated in glycolytic cancer cells whereas MCT4 is up-regulated in the surrounding fibroblast cells which fuel cancer growth [25,26]. The expression of MCT1 has also been found to correlate with the malignancy of cancer cells [27]. In the present study using 3D cultures, MDA-MB-231 cultured alone expressed MCT1 (Figure 5A,B). MDCK cultured alone also expressed MCT1 faintly (Figure 5C,D). When these two cell types were co-cultured, however, the MCT1 expression was diminished to an undetectable level in both MDA-MB-231 and MDCK (Figure 5E,F). Therefore co-culture with MDCK appears to alter the energy consumption pattern of MDA-MB-231 cells, whereby the intake of monocarboxylates is reduced, thus perhaps, mimicking the metabolism pattern of less malignant cells.

MCT4 is expressed by cancer-associated fibroblasts [25] as well as by MDA-MB-231 cells [28]. In the culture conditions of the present study, MDA-MB-231 exhibited MCT4 expression on their cell membrane in the 3D culture, as well as in 2D as reported previously [28], and no clear difference was observed by adding MDCK cells to the culture (data not shown). However, it was noticed that the MCF7 cells expressed a very low level of MCT4 in 2D culture in contrast to a relatively strong level of MCT4 expression in the 3D culture (Figure 5G-J). The implication of this result is not clear; however, it revealed a substantial difference in the metabolic pattern of a breast cancer cell line depending on the culture condition and therefor highlights the importance of utilizing both 2D and 3D culture when investigating cancer cells.

The result showing a change in the expression of MCT1 by MDA-MB-231 cells due to the presence of MDCK cells demonstrates that the effect of the normal epithelium on cancer cells is not limited to cellular behaviour as shown in the time-lapse movies; it also effects on the metabolism and protein expression profile.

### The effect of co-culture on the secretion of MMP2 by MDA-MB-231 cells

**Figure 6:**
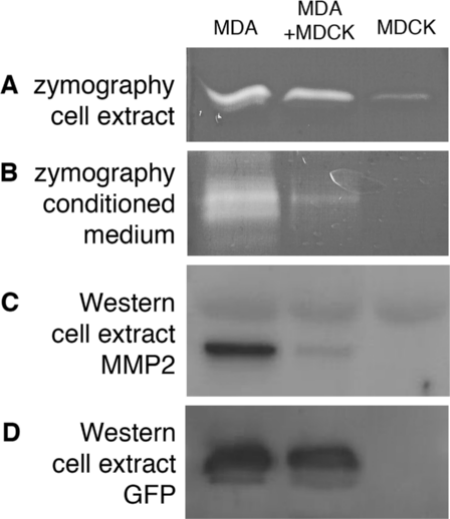
**Zymography and Western blot analyses after co** **-culturing MDA-** **MB** **-231 with MDCK. (A)** Zymography analysis of cell extracts from an MDA-MB-231 monoculture (MDA), a co-culture of MDA-MB-231 and MDCK (MDA + MDCK), and an MDCK monoculture (MDCK). The amount of MDA-MB-231 cells present was kept the same in the first two lanes (i.e. the MDA and MDA + MDCK lanes). The amount of MDCK cells was also kept the same in the last two lanes (i.e. the MDA + MDCK and MDCK lanes). Hence, the total amount of cells in the MDA + MDCK lane is the sum of the amount of cells in each monoculture (MDA and MDCK lanes). **(B)** Zymography analysis of conditioned media from the same cultures as in **(A)**. **(C)** Western blot detecting MMP2 from cell extracts obtained in the same way as A. In **(A-C)**, the MDA + MDCK samples show less gelatinase activity **(A, B)** and less MMP2 expression **(C)** compared to the additive amount of MDA alone and MDCK alone. **(D)** Western blot detecting GFP from the same blot as C, showing the similar amount of MDA-MB-231 cells in the first two lanes.

Extracellular matrix functions as a gatekeeper against metastatic growth [29]. Proteolysis by tumour cells breaks down extracellular matrix, thus facilitating cell growth and migration [30]. It was empirically noticed in this study that the Geltrex® matrix containing MDA-MB-231 cells became fragile after a week of culture, especially in the culture of MDA-MB-231 cells alone, compared to that containing both MDA-MB-231 and MDCK cells. MDA-MB-231 cells are known to secrete metalloproteinases (MMPs) including MMP2, which promote degradation of the basal lamina and extracellular matrix *in vivo* and therefore assist the invasive movement [31–33]. Based on the intact nature of the matrix in the co-culture of MDA-MB-231 with MDCK compared to the fragile nature of the matrix with MDA-MB-231 culture alone, reduced expression of MMPs by MDA-MB-231 in the presence of MDCK was suspected. Gelatin zymography revealed that gelatinase activity was reduced, both in the cell extract and conditioned media when MDA-MB-231 cells were co-cultured with MDCK, compared to the gelatinase activity of the same amount of MDA-MB-231 cells cultured alone (Figure 6A,B). Western blotting further revealed a decrease in MMP2 (gelatinase A) when MDA-MB-231 cells were co-cultured with MDCK (Figure 6C,D), supporting the notion of attenuated proteolytic activity in the presence of MDCK cells.

## Discussion

By using 3D cultures, this study demonstrates the dynamic behaviour and interaction of MDA-MB-231 cells with normal epithelial cells, which cannot be seen in conventional 2D cultures. The MDA-MB-231 cells cultured alone exhibit either spindle- or round-shapes but can interchange between these two shapes in a matter of hours (Figure 1A-E). In co-cultures with epithelial cells which form spheres in 3D, the MDA-MB-231 cells tend to surround and engulf the epithelial mass over time (Figure 2). This results in a loss of spherical structures in the epithelia, cytoplasmic re-distribution of f-actin and β-catenin, and eventual degradation of the cells (Figure 3). The MDA-MB-231 cells can approach the epithelial spheres from a distance (Figure 2D). Regardless of whether there are epithelial cells nearby, the MDA-MB-231 cells tend to engage with one sphere at a time, leaving other nearby epithelial spheres intact (Figure 3C). The MDA-MB-231 cells effectively surround, engulf and destroy the epithelial cells when the MDA-MB-231 cells outnumber the epithelial cells (Figure 3F). On the other hand, if there is a relatively small population of MDA-MB-231 compared to the amount of epithelial spheres, the MDA-MB-231 cells do not engulf the epithelial spheres, despite repeated contacts (Figure 3G-I). The unique features of cellular behaviour which were revealed in this time-lapse study are; (1) the dynamic cell morphology and movement of MDA-MB-231 cells, (2) phagocytosis, (3) population dominance-dependent cell-cell interaction and (4) attraction of the cancer cells to the normal epithelial cells. Furthermore, changes in the protein expression of the MDA-MB-231 cells due to the presence of normal epithelial cells were also demonstrated, in the view of the metabolism pattern of the cancer cells.

### Dynamics in cell morphology and movement

Metastatic cancer cells generally exhibit spindle-shape morphology in 2D, reflecting their invasive phenotype *in vivo*. However, malignant tumours *in vivo* have different microenvironments in the core and peripheral parts of the tumour [34] and only the cells at the periphery of the tumour are readily motile. Although it is not clear as to how much our 3D culture system accurately reflects the microenvironment of micro tumors *in vivo*, it is worth noting that the MDA-MB-231 cells readily change their morphology from the round shape to spindle shape, and vice versa. In addition, the individual cells which form a mass move actively within the mass (Additional file 8: Movie 8). These findings were only possible by using 3D time-lapse studies.

In addition, to the best of our knowledge this is the first study to describe MDA-MB-231 cells changing location while maintaining the overall structure of the mass, i.e. a mass of MDA-MB-231 cells can move as a group (Additional file 3: Movie 3). Given that the cells in the mass actively move within the mass (Additional file 8: Movie 8), the movement of the individual cells may provide the driving force for the rotation as well as translocation of the tumour. It has yet to be determined, however, whether this translocation method is in fact employed *in vivo* in the local expansion of tumours.

3D culture in this study revealed a wide variation in the morphology of individual cells. While it has been shown to be dynamic, the variety of morphology in 3D may also reflect heterogeneity within the cell line. As reported previously, the heterogeneity is not limited to the cell morphology; for example, the MDA-MB-231 cell line is a mixed population in light of the expression of cell surface markers CD24 and CD44, where CD44^+^/CD24^-/low^ cells are the majority and are mixed with CD44^+^/CD24^+^ and CD44^-^/CD24 [35–37]. Although it is uncertain as to whether there is any dynamism in the expression of these markers, there appears some degree of heterogeneity within the cell line.

### Phagocytic activity of cancer cells

Having surrounded the normal epithelial cells, the MDA-MB-231 cells then appeared to exhibit phagocytic activity on the normal cells (Figure 3D,E). Small vesicles labeled with mCherry, indicating their MDCK-origin, were found in the cytoplasm of the MDA-MB-231 cells (Figure 3E, Additional file 9: Movie 9). Transmission electron microscopic studies also revealed multiple vesicular inclusions in the MDA-MB-231 cells that were co-cultured with MDCK (Figure 4G). This poses the question ‘are MDA-MB-231 cells able to act as phagocytes?’ Previous studies have reported that mammary epithelial cells (the origin of MDA-MB-231) have the potential to function as phagocytes, in order to clear up apoptotic alveolar epithelial cells after lactation and weaning [38]. In addition, there are reports on the phagocytic activity of cancer cells, such as breast cancer cells MCF7 on candida albicans [39] and lymphoid neoplasm-derived cells acquiring phagocytic activity after many passages [40]. More recent studies have highlighted functional similarities between macrophages and metastatic cancer cells, suggesting the origin of metastasis to be a fusion of macrophage and neoplastic cells [41,42]. These findings support our interpretation of the phenomenon of engulfing and digesting of epithelial cells by MDA-MB-231 cells to be phagocytic activity.

### Population dominance

This study showed that the ratio of cells used in the co-cultures of MDA-MB-231 and MDCK cells had a strong impact on cellular behaviour and survival. When the MDA-MB-231 cells were in the majority, the engulfing effect was prominent and hence most of the MDCK cells were found as debris after 10 days of culture (Figure 3D-F; Additional file 9: Movie 9). On the other hand, when the MDA-MB-231 cells were in the minority, the engulfing behaviour was rarely observed. Instead the MDA-MB-231 cells approached the MDCK cells, made contact, and then either maintained the contact or moved away (Figure 2G, 3G-I; Additional file 7: Movie 7, Additional file 11: Movie 11). It seems that the MDA-MB-231 cells are more likely to maintain contact with the MDCK cells when a substantial number of MDA-MB-231 cells are already attached to the surface of the MDCK cells (Figure 3B, Additional file 10: Movie 10; also compare the first 12 hours and last 10 hours of Additional files 7: Movie 7). Consequently, once a small number of MDA-MB-231 cells successfully attach and colonise on the surface of the MDCK cells, the MDA-MB-231 population is further augmented, resulting in population dominance by MDA-MB-231.

The effect of co-culture of cancer and normal epithelial cells on the growth of the cells has also been reported in other combinations of cells. Co-culture with an increased amount of normal breast epithelial cells causes a reduction in cell proliferation of many breast cancer cell lines [5]. In another example, epidermal growth factor (EGF), which markedly promotes the cell growth of normal epithelial cells and only moderately promotes the growth of cancer cells, actually inhibits the growth of cancer cells when both normal and cancer cells are co-cultured [6]. Thus, the rapid growth of normal epithelial cells hinders the growth of cancer cells, and once the population balance shifts to the normal epithelial cells, they gain dominance.

The phenomenon of cell ratio affecting cancer cell growth may reflect what happens in metastasis *in vivo*. When a metastatic cancer cell or cells arrive in the new host environment, the initial growth is attenuated by paracrine-mediated growth inhibition from epithelial and mesenchymal cells, hence successful initiation of cell division, colony formation and growth as a secondary tumor is very inefficient [1,10,43]. Our finding that the MDA-MB-231 cells were not as aggressive towards the MDCK cells when the MDA-MB-231 cells were in the minority (Additional files 7: Movie 7, Additional file 10: Movie 10) may reflect the reduced survival rate of cancer cells in a secondary location *in vivo*. However, once cancer cells overcome the self-defense mechanism, they can grow rapidly, which may be reflected by our findings of successful growth of MDA-MB-231 in a co-culture with MDCK where the cancer cells were in the majority (Figure 3D-F; Additional file 6: Movie 6, Additional file 9: Movie 9).

### Attraction of cancer cells towards normal epithelial cells

Another significant finding from our time-lapse studies was that the MDA-MB-231 and MDCK cells were attracted to each other from a distance (Figure 2D,E; Additional file 4: Movie 4, Additional file 5: Movie 5). This resulted in most of the MDA-MB-231 cells attaching to the basal side of the epithelia over time (Figure 2A, 3B). Perhaps this approach of the MDA-MB-231 cells towards the MDCK cells from the basal side is most relevant in the scenario where metastatic cancer cells reach normal epithelial cells at the secondary site. It is intriguing that the MDA-MB-231 cells were attracted more efficiently when there were other MDA-MB-231 cells around (Figure 3C). It has yet to be confirmed whether chemoattraction is involved in this process and, if so, what specific factors are involved. Whether or not there is a community effect among the cancer cells that enhances the efficacy of migration also remains to be established.

### Morphological changes in epithelial cells due to the presence of cancer cells

It was revealed that interaction of the MDA-MB-231 cells with the basal surface of the MDCK spheres resulted in deformity of the epithelial sphere (Figure 3B; Additional file 10: Movie 10), as well as cytoplasmic re-distribution of β-catenin in the epithelial cells (Figure 3B). This suggests that physical force exerted by the cancer cells may disrupt the apical-basal polarity and possibly activate the Wnt/β-catenin pathway in the normal cells. This assumption is based on our previous work which suggested that cellular remodeling processes such as epithelial-mesenchymal transition sufficiently activates the Wnt/β-catenin pathway, concomitant to the accumulation of β-catenin in the cytoplasm [44]. These findings highlight the possible effect of cancer cells on normal cells – cancer cells not only physically compress the normal cells, but also affect intracellular signal transduction in normal cells.

### Changes in the metabolic pattern of MDA-MB-231 cells when co-cultured with normal cells

The metabolic preference to consume glucose and utilise mitochondrial function has attracted attention in cancer research for generations [45]. Oncogenic signal transduction cascades often target mitochondria, and dysfunction of mitochondria may even lead to cancer [46]. On the other hand, when cancer cells adopt a differentiated state thus losing their malignancy, the number of mitochondria in the cells increases [47]. In agreement with these findings, our electron microscopic analyses demonstrated that MDA-MB-231 cells cultured alone showed few mitochondria and few organelle in the cytoplasm (Figure 4D), whereas MDA-MB-231 cells co-cultured with and attached to MDCK cells had a relatively large number of mitochondria (Figure 4F). This finding, together with the reduction of MCT1 expression in MDA-MB-231 cells due to the presence of MDCK cells (Figure 5A-F), suggests that MDA-MB-231 cells change their metabolic pattern, in the presence of MDCK cells, to that of a less malignant phenotype.

## Conclusions

Altogether, this work demonstrates the impact of the presence of normal epithelial cells on cancer cells. Many of the cancer cell dynamics described in this study only became evident by using 3D culture. This culture method is a powerful technique to assess cancer cell dynamics, particularly in the aspect of motility and the effect on normal host cells. A variety of cell combinations will address the effect of different cell types on cancer cells. As this 3D culture method does not require cell passage for at least two weeks, it is therefore suitable for primary cells as well. Possible applications of these techniques in the future include addressing the cellular response to drugs and the organ-specific affinity of cancer cells.

## Methods

### Cell culture

MDA-MB-231 and MCF7 from ATCC were labeled with GFP by lentiviral infection following preparation of the virus using pGIPZ transfection (Open Biosystems) on HEK293T cells following manufacturer’s instructions. To establish MDCK cells stably expressing mCherry-CAAX, MDCK cells were transfected with pcDNA6 mCherry-CAAX using Lipofectamine™ 2000 (Life Technologies) according to manufacturer’s instructions, followed by selection in medium containing 5 μg/ml of blasticidin. These cell lines were cultured in DMEM (Sigma D5546) in the presence of 10% FCS. MCF10A were cultured in DMEM/F12 with supplements as described [21]. All cells were maintained in 2D in plastic dishes until the 3D cultures were set-up.

### Live cell imaging

Co-cultures of MDA-MB-231 and MDCK cells were set up as follows. 30 μl of Geltrex™ reduced growth factor basement membrane matrix (Gibco 12760-021) was spread onto 35 mm μ-dishes (Ibidi 81151) and allowed to set at 37°C for twenty minutes. Cells grown in 2D plastic dishes were dissociated and diluted at 1×10^5^ cells/ml in the culture medium. Various ratios of cells were used; MDA-MB-231 alone, MDA-MB-231:MDCK =20:1, 5:1, 1:1 or 1:20. MCF7 cells with MDCK or MCF10A cells were also used at a ratio of 1:1. 300 μl of cell dilution containing 3×10^4^ cells in total was put into 1.5 ml eppendorf tubes, centrifuged and resuspended in 30 μl of Geltrex™. The mix was then pipetted on top of the set Geltrex™. The plate was then incubated at 37°C for twenty minutes to allow the matrix to set. 500 μl of growth medium was then added to each well. When MDA-MB-231 and MCF10A were co-cultured, the growth media for each were mixed at the ratio of 1:1. The medium was replaced every three days and the cells were grown for 7-12 days. The multi-dimensional microscopy was employed using the Andor Revolution Laser Confocal spinning disc microscope system equipped with the inverted Nikon Ti microscope, Motorised XYZ Prior stage and Andor iXon897 EM EMCCD camera operated by Andor IQ2.6 software.

The experimental setup included sequential time-lapse acquisition (every 1-20 minutes) from 10-40 different X-Y fields, in two fluorescent channels, GFP and mCherry. For each time point and X-Y location, a Z-stack of 9-40 optical sections has been acquired, except for Additional file 1: Movie 1, Additional file 2: Movie 2, Additional file 3: Movie 3 that had no Z-stack. Resulting datasets were split by X-Y positions and the 3D reconstruction was analysed using Imaris 7.6 software. For Additional file 4: Movie 4, Additional file 5: Movie 5, Additional file 6: Movie 6, the 3D files underwent the blind deconvolution by Autoquant 3.0.

### Transmission electron microscopy

3D cultures of; MDA-MB-231 cells alone, MDCK cells alone, and a mix of MDA-MB-231: MDCK = 2:1 in the Geltrex, were fixed with 2.5% glutaraldehyde in Sorensen phosphate buffer, pre-warmed to 37°C, for 2 hours at room temperature and then overnight at 4°C. This was followed by treatment with 1% OsO_4_, dehydration with ethanol and embedding in EPON. Ultrathin sections were cut using Leica 6 ultramicrotome and images were acquired using FEI Tecnai 12 transmission electron microscope at 120 kV acceleration at room temperature.

### 3D culture for immunostaining

Co-cultures of MDA-MB-231 or MCF7 with MDCK or MCF10A cells were set up as above, except that 12 mm-diameter glass coverslips in 4-well plates were used instead of 35 mm μ-dishes. After being incubated for 7-12 days, the samples were fixed in 4% paraformaldehyde (PFA) in PBS for 30 minutes on ice and processed for immunostaining. The primary antibodies used were anti-GFP (Invitrogen, A6645), anti-vimentin (Abcam Ab8978-100), anti-MCT1 (Santa Cruz, Sc-14916), anti-MCT4 (Santa Cruz Sc-50329), anti-smooth muscle actin (Sigma A2547) and anti-β-catenin (BD 610153). After incubating with the appropriate primary and secondary antibodies, cell nuclei were stained with DAPI (Invitrogen S36938) before mounting with mowiol 4-88 (Sigma 81381).

### Gelatinase zymography and Western blot

3D co-cultures were set up for zymography using 3×10^4^ cells of MDA-MB-231, 2×10^4^ cells of MDCK, or a mix of 3×10^4^ cells of MDA-MB-231 and 2×10^4^ cells of MDCK in 4-well plates as described above and cultured for 10 days. The cells were collected along with the geltrex by scraping with 50 μl of cell extraction buffer consisting of 150 mM NaCl, 20 mM TrisHCl pH7.5, 1% Triton X-100 and 1 mM 4-(2-Aminoethyl) benzenesulfonyl fluoride hydrochloride. Conditioned media were collected from similarly cultured groups of cells grown in 5% Geltrex diluted in medium. The medium containing 5% Geltrex was replaced two days after the culture and was then collected for zymography analyses seven days later. Zymography analyses were performed using 10% acrylamide running gels with 1 mg/ml gelatin as described [48]. For Western blotting, co-cultures were set up using 5×10^5^ MDA-MB-231 cells, 4×10^5^ MDCK cells, or a mix of 5×10^5^ MDA-MB-231 cells and 4×10^5^ MDCK cells in 35 mm diameter culture dishes and incubated for seven days. Cell extracts were collected using 100 μl of the above cell extraction buffer and Western blot analyses were carried out using anti-MMP2 (Abcam AB79781) antibody. Anti-GFP (Invitrogen A6645) was used to detect MDA-MB-231 cells exclusively.

## Additional files

Additional file 1: Movie 1. 3D monoculture of MDA-MB-231 cells (green), showing that cells in the aggregate dissociate and become spindle-shaped. Figure 1C shows snapshots of the top-half area of this movie. Many spindle-shaped cells become round and vice versa in the bottom-left area. The large mass in the upper half of the field shows active dissociation of cells. A mass of cells on the right side of the field appearing in the second half of the film shows movement of round cells within the mass. The images were captured every minute at a single Z-plane. Duration of filming was 14 hours and 53 minutes. Additional file 2:
**Movie 2.** 3D monoculture of MDA-MB-231 cells (green), showing dynamic interchange of round and spindle morphology of individual cells. The left lower side of the central mass presents active movement of both spindle and round cells. Figure 1D shows snapshots of the left-hand-side area of this movie at the 6:00-10:00 hour time frames. The images were captured every minute at a single Z-plane. Duration of filming was 14 hours and 53 minutes. Additional file 3:
**Movie 3.** 3D monoculture of MDA-MB-231 cells (green), showing that round cells and small aggregates are able to move and change location in the matrix. Figure 1E shows snapshots of this movie, focusing on the aggregates near the centre. At the 6:00 hour time-point, a relatively large aggregate appears in the left side of the field, in which individual cells moving within this aggregate can be seen. This movie was captured at one Z-plane. The images were captured every minute at a single Z-plane. Duration of filming was 14 hours and 53 minutes. Additional file 4:
**Movie 4.** 3D co-culture of MDA-MB-231 (green) and MDCK (red) cells, showing the attraction of the MDA-MB-231 cells toward the MDCK cells from a distance. A protrusion of MDA-MB-231 cell(s) appears at the 7:00 hour time frame, which extends towards the MDCK cells, makes contact with them and then retracts together with the MDCK cells and nearby MDA-MB-231 cells. Figure 2D shows snapshots of the centre area of this movie. This movie is deconvoluted to eliminate background signals. The images were captured every 20 minutes at 21 Z-planes (ΔZ = 4 μm; total Z range, 80 μm). Duration of filming was 16 hours. Additional file 5:
**Movie 5.** 3D co-culture of MDA-MB-231 (green) and MDCK (red) cells, showing an MDCK sphere being attracted towards a stream of MDA-MB-231 cells. The MDA-MB-231 cells continue migrating in both directions in a stream (horizontally in this movie frame). At the 10:40 hour time-point, some MDA-MB-231 cells begin to approach the MDCK sphere. The sphere is then attracted towards the stream of MDA-MB-231 cells. Once the MDCK sphere is attached to the stream of MDA-MB-231 cells (12:40 hour time-point), many MDA-MB-231 cells begin to surround the MDCK sphere. This film is deconvoluted to eliminate background signals. Figure 2E shows snapshots from the centre area of this movie. The images were captured every 20 minutes at 21 Z-planes (ΔZ = 4 μm; total Z range, 80 μm). Duration of filming was 21 hours. Additional file 6:
**Movie 6.** 3D co-culture of MDA-MB-231 (green) and MDCK (red) cells, showing a large mass of MDA-MB-231 cells engulfing small MDCK spheres. MDCK spheres are seen outside of the mass of MDA-MB-231cells at the first time-point (one at the top left and another on the right-hand-side of the mass). These MDCK cells are taken into the mass of MDA-MB-231 cells within 10 hours. Figure 2F shows snapshots from this movie. The images were captured every 20 minutes at 21 Z-planes (ΔZ = 4 μm; total Z range, 80 μm). Duration of filming was 16 hours. Additional file 7:
**Movie 7.** (3 series). 3D co-culture of MDA-MB-231 (green) and MDCK (red) cells, showing interaction of a relatively small number of MDA-MB-231 cells with the MDCK cells. The movie consists of three parts that were taken consecutively. The first (Movie 7-1) was 12 hour 55 minutes, the second (Movie 7-2) was 7 hour 40 minutes and the third (Movie 7-3) was 13 hour 20 minutes. The MDA-MB-231 cells are attracted towards the MDCK sphere, however, they continue attaching and detaching, until the 20 ~ 21 hour time-point (the end of the second movie to the beginning of the third movie) when a substantial group of MDA-MB-231 cells assemble. After that, a group of MDA-MB-231 cells attach firmly and appear to try to recruit more MDA-MB-231 cells from the top right-hand side. Figure 2G shows snapshots of these three movies. The images were captured every 5 minutes at 25 Z-planes (ΔZ = 1.55 μm; total Z range, 37.2 μm). Total duration of filming was 34 hours 20 minutes. Additional file 8:
**Movie 8.** (3 series). 3D co-culture of MDA-MB-231 cells (green) and MDCK (red) cells, showing that the MDA-MB-231 cells surround one sphere of MDCK cells at a time, until that MDCK sphere is fully surrounded, leaving other nearby MDCK spheres intact. Movie 8 consists of three parts that were taken consecutively. The first (Movie 8-1) was 12 hour 55 minutes, the second (Movie 8-2) was 7 hour 40 minutes and the third (Movie 8-3) was 13 hour 20 minutes. At the beginning of the film (0:00 of Movie 8-1), one MDCK sphere is surrounded by MDA-MB-231 cells while the other sphere, immediately below it (the red mass at the centre of the field), is not. This second MDCK sphere is kept intact despite that some MDA-MB-231 cells pass nearby it. During the 3 to 10 hour time-frames in Movie 8-1, the MDA-MB-231 cells appear to cover the second MDCK sphere, however, they are at different focal levels (confirmed by the layer analyses), and hence, they were close-by but did not form a stable contact. Those MDA-MB-231 cells instead join the first MDCK mass. The MDA-MB-231 cells begin to surround the second MDCK sphere only during the second half of the third film (Movie 8-3), when the first MDCK sphere has been completely surrounded by many MDA-MB-231 cells. This movie also shows that, while not interacting with the MDCK cells, the MDA-MB-231 cells in a mass (bottom right) continue moving within the mass (Movie 8-1 and Movie 8-2), thus maintaining motility in the form of a round aggregate, as were seen in the monoculture of MDA-MB-231 cells (Additional file 3: Movie 3, an aggregate on the left-hand-side). Figure 3C shows snapshots of Movie 8. The images were captured every 5 minutes at 25 Z-planes (ΔZ = 1.55 μm; total Z range, 37.2 μm). Total duration of filming was 34 hours 20 minutes. Additional file 9:
**Movie 9.** 3D co-culture of MDA-MB-231 (green) and MDCK (red) cells, showing that MDCK cells which are surrounded by a relatively large number of MDA-MB-231 cells appear to be destroyed and are seen as debris. Occasionally (at the 2:00, 8:20 and 12:40 hour time-points, for example) small red phagosome-like structures are observed in the cytoplasm of the MDA-MB-231 cells. Figure 3E is a snapshot from this movie at the 11:32 hour time point showing the focal plane containing the red MDCK debris in the center of the MDA-MB-231 cells. Figure 3E shows snapshots of the centre area of this movie. The images were captured every 4 minutes at 16 Z-planes (ΔZ = 2 μm; total Z range, 16 μm). Duration of filming was 13 hours 32 minutes. Additional file 10:
**Movie 10.** 3D co-culture of MDA-MB-231 (green) and MDCK (red) cells, showing the MDA-MB-231 cells in continuous contact with the surface of a relatively large mass of MDCK cells. The MDCK mass is slightly deformed by the end of the movie, but is not largely affected. The images were captured every 5 minutes at 31 Z-planes (ΔZ = 1.94 μm; total Z range, 58.2 μm). Duration of filming was 28 hours. Additional file 11:
**Movie 11.** 3D co-culture of MDA-MB-231 (green) and MDCK (red) cells, showing a few MDA-MB-231 cells stuck on the surface of the MDCK sphere without any other MDA-MB-231 cells joining them for more than 10 hours. The MDA-MB-231 cells appear to break into pieces at the 11:00 hour time point. Figure 3I shows snapshots of this movie. The images were captured every 5 minutes at 25 Z-planes (ΔZ = 1.55 μm; total Z range, 37.2 μm). Duration of filming was 12 hours 46 minutes.

## Abbreviations

2D: Two-dimensional
3D: Three-dimensional
MCT: Monocarboxylate transporter
MMP: Metalloproteinase
EGF: Epidermal growth factor
MDCK: Madin-Darby Canine Kidney
PFA: Paraformaldehyde
